# A Warning

**Published:** 1899-07

**Authors:** Montague R. Leverson


### A WARNING.

### A Severe Epidemic Foretold for the Next Year.

We have had two seasons of epidemic influenza, and now a prolonged drought, permitting aerial percolation of the soil to an unusual depth.

One of the few things which has been learned of the nature of epidemics is, that the above conditions are premonitory of a severe epidemic next year. When the soil is saturated with cadaveric poisons, such for instance as prevailed in consequence of the wars and famines and imperfect burials of the Middle Ages, and when towns and country vied in filth and insanitary conditions, the plague, the black death, the sweating sickness, and typhus fever made periodical ravages among an ignorant and filthy people, whom nature took this means of impelling to better behavior.

Sanitary conditions generally have been greatly improved in most parts of Europe and the United States; but the accumulations of cadaverous poisons in the soil which entailed such severe chastisements down to within the past three centuries, have been replaced by a yet more barbarous practice, more inexcusable by far than the soil-poisoning of the Middle Ages. The cadaveric poisons, now absent or nearly so, from the soil have been instead accumulated in the bodies of the races which inhabit Europe and the United States.

For more than a century the poison of diseases of men and beasts have been inoculated into human beings, under the strange hallucination that such a pathogenic element, infiltrating the human organism and finding therein a culture medium among the most fertile in nature, can produce in such organism a capacity for resisting epidemic diseases. We do know, and it is one of the very few laws of pathology which we do know, that toxines and ptomaines and our excreta, and the excreta of other animals are among the factors which produce fevers, inflammations, and infectious diseases, and that their products are to be found in their most concentrated form in the matter of abscesses and the like. We do know, as was truly said by Sir John Simon, that "when a given body is possessed by a metabolic contagion no product of that body can be warranted as safe not to convey the infection," and the pouring of this cadaveric poison into the blood of the race for now several successive generations, must have produced in the race a receptivity to epidemic diseases which the merest spark may raise into a devastating plague. Something of this condition was probably the principal cause of the great pandemic of small-pox in the years 1871-3, and as unfortunately the world which assumes to itself the title of "civilized" failed to read the lesson aright, a new and perhaps more terrible affliction is to be speedily looked for.

Philadelphia has already experienced something of this in a severe epidemic of typhoid fever, now probably temporarily at an end. It is true that it has been attempted to ascribe that epidemic to the bad water obtained from the Schuylkill river. It is by no means certain that the bad water was even an inciting cause of the epidemic, but alone it could not have produced the fever; a predisposing cause was essential, and this was furnished and the community prepared to receive the infection by the inoculation of successive generations with the putrefying matter of disease.

MONTAGUE R. LEVERSON.

